# Effects of *Bacillus coagulans* supplementation on the growth performance and gut health of broiler chickens with *Clostridium perfringens*-induced necrotic enteritis

**DOI:** 10.1186/s40104-017-0220-2

**Published:** 2018-01-25

**Authors:** Yuanyuan Wu, Yujing Shao, Bochen Song, Wenrui Zhen, Zhong Wang, Yuming Guo, Muhammad Suhaib Shahid, Wei Nie

**Affiliations:** 1 State Key Laboratory of Animal Nutrition, College of Animal Science and Technology, China Agricultural University, Beijing, China; 2 College of Biology, China Agricultural University, Beijing, China

**Keywords:** *Bacillus coagulans*, Broiler chickens, *Clostridium perfringens*, Gut health

## Abstract

**Background:**

The poultry industry is in need of effective antibiotic alternatives to control outbreaks of necrotic enteritis (NE) due to *Clostridium perfringens*.

**Methods:**

This study was conducted to investigate the effects of feeding *Bacillus coagulans* on the growth performance and gut health of broiler chickens with *C. perfringens*-induced NE. Two hundred and forty 1-day-old broiler chicks were randomly assigned to a 2 × 2 factorial arrangement with two dietary *B. coagulans* levels (0 or 4 × 10^9^ CFU/kg of diet) and two disease challenge statuses (control or NE challenged).

**Results:**

NE-induced reduction in body weight gain was relieved by the addition of *B. coagulans* into broiler diets compared with the NE-infected birds. NE infection damaged intestinal morphological structure, promoted intestinal *C. perfringens* growth and liver invasion, and enhanced anti-*C. perfringens* specific sIgA concentrations in the gut and specific IgG levels in serum compared with the uninfected birds. NE infection significantly (*P* < 0.05) decreased mucin-2 (at 14 d post-infection (DPI), toll -like receptor 2 (*TLR2*, at 7 and 14 DPI), *TLR4* (at 7 and 14 DPI), tumor necrosis factor super family 15 (*TNFSF15*, at 7 and 14 DPI), lysozyme (*LYZ*, at 14 DPI) and *fowlicidin-2* (at 7 and 14 DPI) mRNA levels, whereas it dramatically (*P* = 0.001) increased *IFN-γ* mRNA levels at 7 DPI. However, challenged birds fed diets supplemented with *B. coagulans* showed a significant (*P* < 0.01) decrease in gut lesion scores, decreased *C. perfringens* numbers in the cecum and liver, and an increase in fowlicidin-2 mRNA levels in compared with the uninfected birds. In addition, compared with the non-supplemented group, dietary inclusion of *B. coagulans* improved intestinal barrier structure, further increased specific sIgA levels and alkaline phosphatase (IAP) activity in the jejunum, enhanced the expression of jejunum lysozyme mRNA, and inhibited the growth, colonization, and invasion of *C. perfringens*; in contrast, it reduced serum-specific IgG concentrations and jejunum *IFN-γ* mRNA levels.

**Conclusion:**

These results indicated that dietary *B. coagulans* supplementation appeared to be effective in preventing the occurrence and reducing the severity of *C. perfringens*-induced NE in broiler chickens.

## Background

The limitation in the use of antibiotics growth promotors (AGP) in the poultry industry has resulted in an increase in specific diseases, such as avian necrotic enteritis (**NE)** [1]. Clinical avian NE is characterized by diarrhea, necrotic intestinal lesions, severe morbidity and mortality, and decreases in growth rate and feed efficiency, whereas subclinical NE is associated with production losses due to poor digestion and absorption, reduced growth rate and feed efficiency, depression, and ruffled feathers [2, 3]. Therefore, the development of effective AGP alternatives to control future NE outbreaks due to *Clostridium perfringens* in the poultry industry is imperative. Possible alternatives to in-feed antibiotics include probiotics, prebiotics and organic acids, or the development of vaccines targeted against *C. perfringens*-derived toxins, including NetB [3].

Probiotics are defined as living or dead microorganisms that positively influence the health of the host when ingested in sufficient amounts [4, 5]. Several studies have shown that probiotics possess various biological functions, including improving nutrient digestibility and intestinal morphological structure and epithelial barrier integrity, maintaining intestinal microbiota balance, defending against enteropathogen adhesion and invasion, and modulating host cellular and humoral immunity [6–8].

Broiler dietary supplementation with one or several beneficial bacteria has proven to be efficient in preventing the overgrowth of pathogens and development of disease [4, 9, 10]. Moreover, a large number of studies has suggested that microbes and their direct products are effective against avian pathogenic *C. perfringens*, *Salmonella*, and pathogenic *Escherichia coli*, and most of these beneficial strains, belong to the genera *Bacillus, Lactobacillus,* and *Enterococci*, and yeast [11–15]. Therefore, probiotics may provide a potential alternative to the prophylactic use of drugs for NE prevention in poultry [7].

*B. coagulans* is a safe, unique, gram-positive, spore-forming, microaerophilic, lactic-acid producing bacterium that does not encode enterotoxins. It possesses a protective, spore-like protein coating that allows it to survive stomach acids and reach the small intestine to germinate and multiply [16]. Hence, it has been used as a probiotic in pig [17], poultry [18–21], cattle [22], and fish [23, 24] production. Previous studies have shown that *B. coagulans* modifies the gastrointestinal microbiology ecology by replenishing the quantity of desirable obligate microorganisms and antagonizing the pathogenic microbes [17, 25], promoting growth performance, and increasing feed digestibility by secreting enzymes such as protease, α-amylase, xylanase, and lipidase and producing amino acids and vitamins [19–21]. Administration of *B. coagulans* strengthens immune responses [26, 27] and reduces gut inflammation [28–32]. In vivo studies also indicate that *B. coagulans* exhibits antimicrobial, antiviral, and antioxidant activity by modulating cytokines, inhibiting reactive oxygen species, and enhancing phagocytosis [27, 33–36]. In vitro studies have also shown that *B. coagulans* has the ability to produce bacteriocin, bacteriocin-like substances (coagulin), and L-lactic acids with broad activity against various strains of *Salmonella,* coliforms, *Listeria monocytogenes,* and *Clostridium* species [37–41]. Thus, *B. coagulans* has the potential to control farm animal pathogens. However, no study has investigated the function of *B. coagulans* in preventing NE infection in broiler chickens to date.

Therefore, the objective of the present study was to evaluate the efficacy of *B. coagulans* in controlling NE infection in broiler chickens by determining its effect on growth performance, *C. perfringens* cecal colonization*, C. perfringens* liver invasion, intestinal morphological changes, gut lesion scores, and immune responses in broilers with *Clostridium perfringens*-induced NE.

## Methods

### Experimental design, birds, and diets

A 2 × 2 factorial arrangement of treatments was employed in a completely randomized design to investigate the effects of two levels of *B. coagulans* supplementation (0 or 4 × 10^9^ CFU/kg of diet) and two levels of NE challenge (challenged or unchallenged). Two hundred and forty 1-day-old male broiler chicks were obtained from a commercial hatchery (Beijing Arbor Acres Poultry Breeding Company, Beijing, China). Upon arrival, the birds were weighed after hatching and randomly assigned to one of the four treatments. Each treatment group had six replicate cages with 10 birds per cage. The treatment groups were as follows: (i) negative control group (neither *B. coagulans* treatment nor NE infection), (ii) the *B. coagulans*-treated group (*B. coagulans* treatment but without NE infection), (iii) NE-infected control group (NE infection but without *B. coagulans* treatment), and (iv) the *B. coagulans*-treated and NE-infected group (both *B. coagulans* treatment and NE infection). *B. coagulans* used in this study was provided by Lifeed Biological Co., Ltd. (Zhuzhou City, Hunan Province, China) at a density of 1 × 10^10^ CFU/g. To avoid cross-contamination, the uninfected birds and NE-infected birds were reared in two separate rooms equipped with three-tiered battery cages (1.0 m × 0.70 m × 1.75 m, length × width × height) with a raised wire-netted floor, respectively. Stocking density was set at 0.07 m^2^ per bird. The room temperature was maintained at 32 °C to 34 °C during the first 5 d and then gradually decreased by 2 °C/wk to reach a final room temperature of 22 °C to 24 °C. The birds received continuous light for the first three day and then maintained under 23 h L: 1 h D for the remainder of the study. In addition, the chickens were vaccinated against Newcastle disease virus (NDV) and infectious bronchitis virus (IBV) vaccines on d 7 and 21, respectively, and against bursa disease virus (IBDV) according to the routine immunization program via drinking water on d 12 and 26.

Antibiotic-free and coccidiostat-free corn-soybean meal-based mash diets were formulated to meet or exceed National Research Council (1994) requirements for starter (d 1 to 21) and grower (d 22 to 42) periods. The composition of the basal diet and nutrient levels are presented in Table 1. The experimental diet was formulated by mixing the basal diet with 400 mg of *B. coagulans* (1 × 10^10^ CFU/g of the product) to reach 4 × 10^9^ CFU/kg of diet. To ensure the homogeneity of the additives, approximately 5 kg of the basal diet mixed with the additive were thoroughly mixed using a plastic bucket. All birds were allowed ad libitum access to feed and water throughout the study.

**Table 1:** Composition and nutrient levels of the experimental basal diet, as-fed basis unless stated otherwise, %

Items	1 to 21 d	22 to 42 d
Composition, %		
Corn	54.65	60.71
Soybean meal	37.35	31.96
Soybean oil	3.52	3.28
Limestone-calcium carbonate	1.11	1.15
Calcium hydrogen phosphate	2.10	1.63
Sodium chloride	0.30	0.30
DL-Methionine, 98%	0.20	0.20
L-Lysine HCl, 78%	0.23	0.23
Vitamin premix^a^	0.03	0.03
Mineral premix^b^	0.2	0.2
Choline chloride, 50%	0.26	0.26
Ethoxyquin, 33%	0.05	0.05
Total	100	100
Calculated Nutrient levels^c^		
Metabolizable energy, MJ/kg	12.33	12.54
Crude protein, %	210.0	190.1
Calcium, %	10.0	9.0
Available phosphorus, %	4.8	4.0
Lysine, %	11.5	10.4
Methionine, %	5.01	4.08

^a^Vitamin premix provided per kilogram of complete diet: vitamin A 9,500 IU; vitamin D_3_ 2,500 IU; vitamin E 30 IU; vitamin K_3_ 2.65 mg; vitamin B_12_ 0.025 mg; biotin 0.30 mg; folic acid 1.25 mg; nicotinic acid 50 mg; *D*-pantothenic acid 12 mg; pyridoxine hydrochloride 6.0 mg; riboflavin 6.5 mg; thiamine mononitrate 3.0 mg

^b^Mineral premix provided per kilogram of complete diet: iron 80 g; copper 8 mg; manganese 100 mg; zinc 80 mg; iodine 0.35 mg; selenium 0.15 mg

^c^Calculated value based on the analyzed data of experimental diets

### NE disease model

*C. perfringens* type A CVCC52 (China Veterinary Culture Collection Center, China Institute of Veterinary Drug Control, Beijing, China) was stored in fluid thioglycollate medium (CM801; Beijing Land Bridge Technology Co., Ltd.) containing 30% (v/v) glycerol, at −20 °C until further use. Approximately 1 mL of the seed stock was cultured in 250 mL of thioglycollate broth for approximately 18 h at 39 °C; 1 mL of the above culture was then inoculated into 250 mL of cooked meat medium supplemented with dried meat particles (CM605; Beijing Land Bridge Technology Co., Ltd.) and incubated for approximately 18 h at 30 °C; and 1 mL of the above culture was inoculated into 1 L of thioglycollate broth that was fortified with 10 g/L of starch and 15 g/L of peptone and incubated for approximately 18 h at 39 °C.

Subclinical NE was induced in broiler birds as previously described with minor modifications [42]. At 14 d of age, each bird in the challenged groups was orally inoculated with a 20-fold dose of attenuated coccidial vaccine (containing live attenuated oocysts of *Eimeria acervulina* PAHY strain, *E. maxima* PMHY strain, and *E. tenella* PTMZ strain; Foshan Standard Bio-Tech Co., Ltd., Foshan, China). Uninfected control birds received 1 mL of sterile PBS instead of the vaccine. All *Eimeria* oocyst-inoculated birds were then subsequently gavaged orally with 1 mL of *C. perfringens* (10^9^ CFU/mL) per day on d 18, 19, and 20. The uninfected birds were gavaged orally with 1 mL of sterile thioglycollate broth synchronously. Feed was withdrawn 8 h prior to each inoculation.

### Growth performance

The feed intake and body weight (BW) of the chickens in each pen were measured on d 1, 14, 21, 28 and 42, and feed intake was recorded during d 1 to 14, d 15 to 21, d 22 to 28, and d 29 to 42. The average feed intake (AFI), body weight gain (BWG), and feed conversion ratios (FCR) were calculated at different experimental periods.

### Intestinal NE lesion scoring and sample collection

At 7 d post *C. perfringens* infection (DPI; at 28 d of age), one bird per replicate was randomly selected, weighed, and euthanized by cervical dislocation. The duodenum, jejunum, and ileum were collected and scored for NE gut lesions on a scale of 0 (none) to 4 (high) by three independent observers who were blinded to the experimental design, as previously described [43]. At 7, 14 and 21 DPI (at 28, 35, and 42 d of age), approximately 1 cm long jejunal samples between Meckel’s diverticulum to the proximal of the jejunum were snap-frozen in liquid nitrogen and stored at −80 °C until further mRNA analysis. Approximately 2 cm long jejunal samples in length midway between the endpoint of the duodenal loop and Meckel’s diverticulum were collected and then flushed and fixed with 10% neutral buffered formalin solution for histological examination. The cecal contents were collected and frozen immediately at −40 °C until further analysis for bacterial populations.

### Gut morphology analysis

Gut morphology and goblet cell analysis was performed as previously described [44]. The fixed tissue samples were dehydrated in a tissue processor (Leica Microsystems K. K., Tokyo, Japan) and embedded in paraffin wax. Paraffin sections (5 μm) were sliced using a microtome (Leica Microsystems K. K.) and mounted on glass slides. The paraffin was removed by xylene (two times for 5 min each), followed by rehydration in 95% alcohol (5 min), and 50% alcohol (5 min). Sections were stained with haematoxylin and eosin (HE) for villous morphology measurement. Villi height and crypt depth of the stained sections were measured under a microscope at 40× combined magnification and an ocular micrometer (Leica Microsystems Ltd., Wetzlar, Germany). Villi height was measured from the tip of the villi to the villi crypt junction. Crypt depth was defined as the depth of the invagination between adjacent villi. Five villi per section and three sections per sample were analyzed.

### Determination of intestinal and liver bacteria concentration

Caecal microflora was analyzed using the culture technique as previously described [44]. Approximately 1 g of each sample (cecal digesta or liver samples) were aseptically collected into pre-weighed 15-mL sterile plastic tubes (Corning Inc., Beijing, China), weighed, and diluted with ice-cold sterile buffered peptone water (CM201, Land Bridge Technology Ltd.) to an initial 10^−1^ dilution and homogenized separately using a Heidolph Diax 600 homogenizer (Heidolph, Schwabach, Germany). The homogenized liver suspension was serially diluted between 10^−1^ to 10^−7^ dilutions, and 100 μL of each diluted sample was subsequently plated on selective agar plates for *C. perfringens* counting in duplicate. The number of *C. perfringens* was determined on tryptose-sulfite-cycloserine agar (TSC, CM 138; Beijing Land Bridge Technology Co., Ltd.) after anaerobic incubation at 37 °C for 48 h (black colonies). Coliform bacteria were plated on MacConkey agar (CM908; Beijing Land Bridge Technology Co., Ltd.) after aerobic incubation at 37 °C for 24 h. The cecal contents were serially diluted in PBS and plated on de Man, Rogosa, and Sharpe agar (MRS agar, CM 188, Land Bridge Technology Ltd.) for enumeration of lactic acid bacteria (LAB) after aerobic incubation at 37 °C for 24 h. *Bifidobacterium* was counted on Bifidobacterium agar (CM194; Beijing Land Bridge Technology Co., Ltd.) after incubation in an anaerobic cabinet at 39 °C for 48 h. The number of colony forming units (CFUs) was expressed as a logarithmic (log_10_) values per gram of intestinal digesta.

### Determination of intestinal alkaline phosphatase activity

Previously frozen jejunal tissue samples were thawed on ice and 350 mg of each tissue was homogenized in 7 mL of 50 mmol/L cold phosphate buffered saline (PBS, pH 7.4) containing 0.5% Triton X-100. The resulting homogenate was then centrifuged at 10,000×*g* (Sorvall Legend 23R, Thermo Fisher Scientific Inc., Waltham, MA, USA) for 15 min, and the supernatant was collected for estimation of protein content (A-045-2, Total Protein Quantitative Assay kit, Nanjing Jiancheng Bioengineering Institute, Nanjing, China) and intestinal alkaline phosphatase (IAP) activity (A-059-2, Alkaline Phosphatase Assay Kit, Nanjing Jiancheng Bioengineering Institute). The assay used stable p-nitrophenol phosphate (pNPP) as substrate. Each sample was normalized to the total protein content to determine specific activity. Total IAP activity was expressed as nmoles pNPP hydrolyzed/min/mg protein.

### Enzyme-linked immunosorbent assay (ELISA) for determining anti-*C. perfringens* specific antibody levels in sera and intestinal mucosa

The level of antibodies against *C. perfringens* was tested using a modified indirect ELISA. *C. perfringens* bacterial lysates were prepared from *C. perfringens* cultures according to a previously published protocol [45]. Briefly, flat-bottomed 96-well microtiter plates (Corning Costar, Corning, NY, USA) were coated overnight at 4 °C with 100 μL/well of whole *C. perfringens* bacterial lysates (10 mg/mL) in 0.1 mol/L carbonate buffer (pH 9.6). The plates were washed thrice with PBS (pH 7.2) containing 0.05% Tween 20 (PBST). The wells were blocked with 200 μL of 1% BSA dissolved in PBS containing 0.05% Tween (PBST) and incubated at 37 °C for 2 h. Then, the plates were washed in PBST and dried at room temperature for 1 h. Approximately 100 μL of the reference serum, serum sample, or intestinal extract was not diluted or diluted at a ratio of 1:50 or 1:4 in PBST with 1% BSA individually and added to wells in duplicate. The plates were incubated at 4 °C overnight and washed thrice with PBST. Diluted horseradish peroxidase-conjugated goat anti-chicken IgG (1:10,000; A30-104P, Bethyl Laboratories Inc., Montgomery, TX, USA) or diluted goat anti-chicken IgA Fc HRP conjugate (1: 20,000; A30-103P, Bethyl Laboratories Inc.) was added to the wells with serum or intestinal supernatant, respectively, and incubated at 37 °C for 2 h. The plates were washed five times with PBST and incubated with 3, 3′, 5, 5′-tetramethylbenzidine (TMB) for 30 min at room temperature in the dark. The reaction was stopped with sulfuric acid (2 mol/L), and the absorbance was measured at a wavelength of 450 nm using an automatic ELISA reader (Bio-Tek EL311SX, Bio-Tek, CA, USA). Each serum or intestinal sample was tested in duplicate.

### Quantitative real-time PCR for measuring immune-related gene transcript levels in the jejunum

Total RNA was isolated from snap-frozen jejunal tissue samples (50 mg) based on the RNeasy mini kit following the animal tissue protocol (Qiagen, Germantown, MD, USA). The purity and concentration of the total RNA were measured in a spectrophotometer (NanoDrop-2000, Thermo Fisher Scientific, Waltham, MA, USA) using the 260:280 nm absorbance ratio. The absorption ratio (OD_260_/OD_280_) of all samples ranged between 1.8 and 2.0. First-strand cDNA was synthesized from 2 μg of total RNA using a Primer Script™ RT reagent Kit with gDNA Eraser (Perfect Real Time; Takara Biotechnology Co. Ltd., Tokyo, Beijing, China) according to the manufacturer’s instructions and stored at −20 °C until further processing. Oligonucleotide primers for chicken *TLR2*, *TLR4*, *TNFSF15*, *IFN-γ*, *LYZ*, *fowlicidin-2*, *mucin-2*, and chicken glyceraldehyde-3-phosphate dehydrogenase (*GAPDH*) (Table 2) were designed based upon sequences available from public databases using Primer Express 5.0 (Applied Biosystems, Foster City, CA, USA) and synthesized by Sango Biotech (Shanghai) Co., Ltd. Primers were designed to span an intron to avoid genomic DNA amplification. Quantitative real-time PCR was performed by Applied Biosystems’ 7500 Fast Real-Time PCR System and a SYBR Premix Ex Taq™ kit (Takara Biotechnology Co. Ltd., Beijing, China). Reactions were conducted in a 20-μL reaction mixture containing 10.0 μL of SYBR Premix Ex Taq (2×) mix, 1.0 μL of cDNA, 0.5 μL of each primer (10 mmol/L), and 8.0 μL of sterile nuclease-free water. For PCR, samples were subjected to an initial denaturation phase at 95 °C for 5 min, followed by 40 cycles of denaturation at 95 °C for 30 s and annealing and extension at 60 °C for 30 s. Melt curve analysis was performed to confirm PCR amplification specificity. All tissue samples used in cDNA synthesis and in the following PCR amplifications were analyzed in triplicate. Gene expression levels of *TLR2, TLR4, TNFSF15, IFN-γ, LYZ, fowlicidin-2*, and *mucin-2* were analyzed with *GAPDH* as endogenous control. The average gene expression of each sample relative to that of *GAPDH* was calculated using the 2^−ΔΔCt^ method [46].

**Table 2:** Sequences of the oligonucleotide primers used for quantitative real-time PCR^a^

Gene	Primer sequence 5′ → 3′	GenBank Accession No.	Efficiency^b^, %
*GAPDH*	*F: GGTGGTGCTAAGCGTGTTAT*	K01485	93.23
	*R: ACCTCTGTCATCTCTCCACA*		
*TNFSF15*	*F: CCTGAGTATTCCAGCAACGCA*	NM010245578	113.59
	*R: ATCCACCAGCTTGATGTCACTAAC*		
*IFN-γ*	*F: AGCTGACGGTGGACCTATTATT*	Y07922	95.29
	*R: GGCTTTGCGCTGGATTC*		
*TLR-2*	*F: CTGGGAAGTGGATTGTGGA*	AB050005.2	93.39
	*R: AAGGCGAAAGTGCGAGAAA*		
*TLR-4*	*F: GGATCTTTCAAGGTGCCACA*	AY064697	98.08
	*R: CAAGTGTCCGATGGGTAGGT*		
*MUC-2*	*F: GGGATATTCACCTGGAGAAACCATT*	XM_421045	100.41
	*R: TTGCATGTCCATCTGCCTGAA*		
*LYZ*	*F: CCCAGGCTCCAGGAACCT*	NM_205281	98.42
	*R: CACGCTCGCTGTTATGTCTGA*		
*Fowlicidin-2*	*F: CAAGGAGAATGGGGTCATCAG*	NM_001605.3	85.54
	*R: CGTGGCCCCATTTATTCATTCA*		

*F* forward, *R* reverse, *GAPDH*, glyceraldehyde-3-phosphate dehydrogenase, *IFN-γ* interferon-γ, *IL* interleukin, *LYZ* lysozyme, *TLR* toll-like receptor, *TNFSF15* tumor necrosis factor super family 15

^a^Primers were designed and synthesized by Sango Biotech (Shanghai) Co., Ltd

^b^PCR amplification efficiency of primers of each gene

### Statistical analysis

The original data was checked by using Grubbs’ test method. If |Xp-$\overline{X}$|>λ (α, n) S, Xp was considered as the outlier. λ (α, n) was obtained from table lookup and S was the standard deviation of that treatment. Afterwards, the data were analyzed using the GLM procedure of SPSS 17.0 (SPSS, Inc., Chicago, IL, USA) as a 2 × 2 factorial arrangement (two levels of *B. coagulans* treatment and two levels of NE challenge). The main effects of *B. coagulans* and NE challenge and the associated two-way interactive effects were analyzed. Duncan’s multiple comparison test was used to separate means when interactive effects significantly differed. The results were expressed as treatment means with their pooled SEM. A *P* value ≤0.05 was considered statistically significant and *P* value between 0.05 to 0.10 was classified as a tendency.

## Results

### Growth performance

The growth performance of the broiler chickens is shown in Table 3. A significant interaction effect (*P* < 0.05) on BWG during d 22 to 28 between *B. coagulans* supplementation and NE infection was observed. NE-induced reduction in BWG was significantly inhibited by the addition of *B. coagulans* into broiler diets compared to that in NE-infected control birds.

**Table 3:** Effect of *Bacillus coagulans* on growth performance of broiler chickens infected with NE

Items	NE^d^	Feed intake, g/d				Body weight gain, g/d				FCR^c^			
		d 1–14	d 15–21	d 22–28	d 29–42	d 1–14	d 15–21	d 22–28	d 29–42	d 1–14	d 15–21	d 22–28	d 29–42
*Bacillus coagulans,* mg/kg													
0	–	347.1	536.2	655.4	2475.0	266.7	316.5	363.0	1344.8	1.30	1.70	1.82	1.84
0	+	358.7	550.3	610.9	2472.0	264.8	316.0	325.6	1341.9	1.36	1.74	1.88	1.85
400	–	346.7	535.2	660.5	2532.0	274.1	341.8	377.2	1431.7	1.27	1.57	1.76	1.77
400	+	345.2	545.8	631.1	2553.0	267.2	339.0	363.9	1398.8	1.29	1.61	1.74	1.83
SEM^e^		3.96	3.95	7.01	27.71	2.91	3.93	7.53	16.74	0.016	0.018	0.034	0.016
Main effect													
400		346.0	540.5	645.8	2542.5	270.6	340.4^a^	370.6	1415.2	1.28	1.59^b^	1.75	1.80
0		352.9	543.3	633.1	2473.5	265.8	316.2^b^	344.3	1343.4	1.33	1.72^a^	1.85	1.84
Challenged		351.9	548.1	621.0^b^	2512.5	266.0	327.4	344.7^b^	1370.3	1.32	1.68	1.81	1.84
Non-challenged		346.9	535.7	658.0^a^	2503.5	270.4	329.1	370.1^a^	1388.2	1.29	1.63	1.79	1.81
*P*-values^f^													
*Bacillus coagulans*		0.402	0.731	0.385	0.237	0.417	0.010	0.036	0.053	0.151	0.004	0.153	0.197
Challenged		0.537	0.142	0.022	0.874	0.460	0.835	0.038	0.603	0.246	0.269	0.771	0.381
Challenged *× Bacillus coagulans*		0.426	0.831	0.600	0.832	0.670	0.889	0.043	0.662	0.673	0.947	0.587	0.463

^a,b^Means within the same column with different superscripts differ significantly (*P* < 0.05)

^c^FCR = feed conversion ratio = g of feed intake / g of BW gain, g/g

^d^NE = Co-challenged with Eimeria spp. and *Clostridium perfringens;* −, without NE challenge; +, with NE challenge

^e^SEM, standard error of the mean

^f^*P*-values represent the main effect of the diet, the main effect of NE challenge, and the interaction between the dietary treatments and NE challenge

Based on the main effect of the challenge, NE challenge only caused a decreased effect (*P* < 0.05) on AFI and BWG during d 22 to 28, whereas no effect on FCR was observed relative to that in uninfected birds.

Based on the main effect of supplementation, dietary *B. coagulans* addition significantly (*P* < 0.05) increased BWG from d 15 to 21 and d 22 to 28, as well as improved FCR from d 15 to 21 compared to that in the non-supplemented group.

### Intestinal morphology and lesion scores

Table 5 shows that there was a dramatic interaction effect (*P* = 0.028) on gut lesion scores between NE infection and *B. coagulans* addition*.* NE-infected birds fed diets supplemented with *B. coagulans* exhibited a significant decrease in gut lesion scores in the small intestine at 7 DPI compared to that in the uninfected birds. However, NE infection significantly decreased villus height to crypt depth ratio (on d 42) and goblet cells number on d 42 (Table 5), and remarkably increased the intestinal lesion scores on d 28 (Table 4) in the small intestines of NE-challenged birds compared to non-infected treatments. A decrease in villus height in the duodenum of NE-infected birds was observed at d 28 and d 42. Furthermore, chickens that received *B. coagulans* diets had greater (*P* < 0.05) villi height (on d 42), higher (*P* < 0.05) villus height to crypt depth ratio (on d 42), and greater (*P* < 0.05) goblet cell numbers (on d 28 and 42) in the jejunum compared to that in the non-supplemented groups (Table 5). Birds fed diets with *B. coagulans* also showed (*P* < 0.05) a reduction in gut lesion scores in the duodenum and jejunum at 7 DPI (d 28) and lower (*P* < 0.05) crypt depth compared to the birds that did not receive *B. coagulans*-supplemented diets.

**Table 4:** Necrotic enteritis (NE) lesion scores in the small intestine of broiler chickens at 7 d after infection treated with dietary supplemental *Bacillus coagulans* and infected with NE^e^

		NE lesion scores			
Items	NE^f^	duodenum	Jejunum	Ileum	Small intestine^h^
*Bacillus coagulans*, mg/kg					
0	–	0.29	0.17	0.17	0.21^c^
400	–	0.21	0.13	0.13	0.15^c^
0	+	1.95	1.48	0.63	1.35^a^
400	+	1.13	0.63	0.50	0.75^b^
SEM		0.097	0.076	0.058	0.083
Main effect					
400		0.67^b^	0.39	0.32	0.45
0		1.13^a^	0.83	0.40	0.78
Challenged		1.54^c^	1.05^a^	0.65^a^	1.05
Non-challenged		0.25^d^	0.15^b^	0.15^b^	0.18
*P*-values^g^					
Challenged		<0.001	<0.001	<0.001	<0.001
*Bacillus coagulans*		0.013	0.074	0.331	0.003
Challenged × *Bacillus coagulans*		0.053	0.198	0.625	0.028

^a, b, c, d^ Means within the same column with different superscripts differ significantly (*P* < 0.05)

^e^Each value represents the mean of 6 birds per treatment (1 bird per replicate). Scores were given from 0 to 4 depending on the severity

^f^NE = Co-challenged with *Eimeria* spp. and *Clostridium perfringens;* −, without NE challenge; +, with NE challenge

^g^*P*-values represent the main effect of the diet, the main effect of NE challenge, and the interaction between the dietary treatments and NE challenge

^h^Small intestine values represent the average of duodenal, jejunal, and ileal scores

**Table 5:** Effect of *Bacillus coagulans* on jejunal morphology and goblet cell numbers of broiler chickens challenged with NE (*n* = 6)

		Villous height, μm		Crypt depth, μm		VH/CD^f^		GC cells^g^	
Items	NE^e^	d 28	d 42	d 28	d 42	d 28	d 42	d 28	d 42
*Bacillus coagulans*, mg/kg									
0	–	1248	1161	281	218	4.65	5.45	44.3	45.5
0	+	1048	1139	294	239	3.65	4.89	42.3	42.0
400	–	1289	1527	241	202	5.99	7.93	52.5	53.3
400	+	1208	1290	233	209	5.41	6.55	53.0	47.0
SEM^h^		34.5	29.8	8.9	7.2	0.292	0.208	1.37	0.68
Main effect									
400		1249	1409^a^	237^b^	206	5.70^a^	7.24^a^	52.8^a^	50.1^a^
0		1148	1150^b^	288^a^	228	4.15^b^	5.17^b^	43.3^b^	43.8^b^
Challenged		1128	1215	264	224	4.53	5.72^d^	47.6	44.5^d^
Non-challenged		1269	1344	261	211	5.32	6.69^c^	48.4	49.4^c^
*P-*values^i^									
*Bacillus coagulans*		0.169	<0.001	0.015	0.141	0.019	<0.001	0.005	0.001
Challenged		0.065	0.051	0.877	0.363	0.196	0.028	0.788	0.004
Challenged ×*Bacillus coagulans*		0.408	0.096	0.551	0.645	0.724	0.306	0.656	0.329

^a, b, c, d^ Means within the same column with different superscripts differ significantly (*P* < 0.05)

^e^NE = Co-challenged with *Eimeria* spp. and *Clostridium perfringens;* −, without NE challenge; +, with NE challenge

^f^VH/CD = villus height to crypt depth ratio

^g^GC cells = goblet cells numbers per mm^2^

^h^SEM, standard error of the mean

^i^*P*-values represent the main effect of the diet, the main effect of NE challenge, and the interaction between the dietary treatments and NE challenge

### Intestinal bacterial colonization and liver *C. perfringens* invasion

An interaction effect on liver *C. perfringens* invasion was observed between NE infection and *B. coagulans* addition (Table 6)*.* Challenged birds fed diets supplemented with *B. coagulans* showed a significantly lower (*P* < 0.05) number of *C. perfringens* in the liver during the entire infection period in contrast to that in the NE-infected birds.

**Table 6:** Effect of *Bacillus coagulans* on intestinal bacterial concentration and liver *Clostridium perfrigens* numbers of broiler chickens challenged with NE (*n* = 6)

		Cecal bacterial counts												Liver *Clostridium perfrigens*		
Items	NE^e^	*Escherichia coli*			*Clostridium perfrigens*			*Lactobacillus*			*Bifidobacterium*					
		d 28	d 35	d 42	d 28	d 35	d 42	d 28	d 35	d 42	d 28	d 35	d 42	d 28	d 35	d 42
*Bacillus coagulans*, mg/kg																
0	–	7.42	7.50	7.63	4.60^c^	4.77	4.75^c^	8.03	8.70	7.95	8.28	8.64	8.40	0.00^c^	0.00^c^	0.00^c^
0	+	7.57	7.77	7.66	7.06^a^	6.87	7.18^a^	8.01	7.48	7.98	8.27	7.61	8.24	3.28^a^	2.71^a^	2.20^a^
400	–	7.30	7.10	7.19	4.39^c^	4.43	4.62^c^	8.44	8.76	8.16	9.05	8.80	8.43	0.00^c^	0.00^c^	0.00^c^
400	+	7.32	7.38	7.40	5.73^b^	5.97	5.72^b^	8.43	8.06	8.20	8.97	8.16	8.43	1.56^b^	1.26^b^	0.69^b^
SEM^f^		0.092	0.044	0.078	0.027	0.043	0.039	0.062	0.091	0.072	0.084	0.076	0.091	0.021	0.022	0.018
Main effect																
400		7.31	7.24^b^	7.30^b^	5.56	5.20^b^	5.16	8.43^a^	8.41	8.81	9.01^a^	8.48^a^	8.43	0.78	0.63	0.35
0		7.50	7.64^a^	7.65^a^	5.83	5.82^a^	5.97	8.02^b^	8.09	7.97	8.27^b^	8.13^b^	8.32	1.64	1.36	1.10
Challenged		7.45	7.58^c^	7.53	6.40	6.67^c^	6.95	8.22	7.77^b^	8.09	8.62	7.88^d^	8.34	2.42	1.99	1.35
Non-challenged		7.36	7.30^d^	7.41	4.50	4.60^d^	4.68	8.23	8.73^a^	8.05	8.66	8.72^c^	8.41	0.00	0.00	0.00
*P-*values^g^																
*Bacillus coagulans*		0.332	<0.001	0.041	<0.001	<0.001	0.001	0.003	0.105	0.152	<0.001	0.032	0.568	0.004	0.007	0.008
Challenged		0.631	0.011	0.457	<0.001	<0.001	0.003	0.911	<0.001	0.785	0.792	<0.001	0.684	<0.001	<0.001	<0.001
Challenged ×*Bacillus coagulans*		0.735	0.934	0.586	0.029	0.051	0.004	0.992	0.173	0.964	0.828	0.234	0.689	0.032	0.017	0.021

^a,b,c,d^ Means within the same column with different superscripts differ significantly (*P* < 0.05)

^e^NE = Co-challenged with *Eimeria* spp. and *Clostridium perfringens;* −, without NE challenge; +, with NE challenge

^f^SEM, standard error of the mean

^g^*P*-values represent the main effect of the diet, the main effect of NE challenge, and the interaction between the dietary treatments and NE challenge

In addition, a significant interaction effect for cecal *C. perfringens* concentration (on d 42) was observed between NE infection and *B. coagulans* addition (Table 6)*.* Challenged birds fed *B. coagulans*-supplemented diets showed lower numbers (*P* < 0.01) of *C. perfringens* in the cecum (on d 42) than that in NE-infected birds.

Compared to the uninfected birds, NE infection significantly (*P* < 0.001) reduced cecal *Lactobacilli* and *Bifidobacterium* counts in chyme on d 35, but significantly (*P* < 0.01) increased cecal *coliform* (on d 35) and *C. perfringens* (both on d 28 and d 42) counts. However, chickens fed dietary *B. coagulans* had lower (*P* < 0.05) *coliform* (on d 35 and d 42) and *C. perfringens* (on d 28, d 35, and d 42) counts in cecal contents, but showed significantly higher (*P* < 0.05) *Lactobacilli* (on d 28) and *Bifidobacterium* numbers (on d 28 and d 35) (Table 6) compared to the un-supplemented groups.

### IAP activity

Table 7 shows that no interaction effect on jejunum IAP activity was observed between *B. coagulans* supplementation and NE infection. However, NE infection showed a decrease (*P* = 0.097) in jejunum IAP activity on d 42 compared to that in non-infected birds. Relative to the broilers fed on a basal diet, those that received *B. coagulans* supplementation displayed a significant (*P* = 0.047) increase in jejunum IAP activity on d 42.

**Table 7:** Effect of *Bacillus coagulans* on serum speicific IgG and intestinal specific IgA against *Clostridium perfringens* and intestinal AKP activity of broiler chickens challenged with NE (n = 6)

Items	NE^e^	sIgA^f^		IgG^g^		IAP^h^	
		d 28	d 42	d 28	d 42	d 28	d 42
*Bacillus coagulans*, mg/kg							
0	–	1.07	1.08	1.80	1.12	455.8	633.7
0	+	1.38	1.35	2.24	1.02	414.4	531.6
400	–	0.98	0.86	1.68	0.81	491.0	733.9
400	+	1.00	1.66	2.31	0.95	471.4	652.5
SEM^i^		0.036	0.103	0.115	0.039	28.94	25.01
Main effect							
400		1.22^a^	1.26	1.99	0.88^b^	481.2	693.2^a^
0		0.99^b^	1.22	2.02	1.07^a^	435.1	582.6^b^
Challenged		1.19^c^	1.51^a^	2.28^a^	0.99	442.9	592.0
Non-challenged		1.02^d^	0.97^b^	1.74^b^	0.96	473.4	683.8
*P-*values^j^							
*Bacillus coagulans*		0.008	0.840	0.910	0.031	0.441	0.047
Challenged		0.045	0.023	0.038	0.801	0.608	0.092
Challenged ×*Bacillus coagulans*		0.069	0.217	0.683	0.131	0.854	0.839

^a,b,c,d^ Means within the same column with different superscripts differ significantly (*P* < 0.05)

^e^NE = Co-challenged with *Eimeria* spp. and *Clostridium perfringens;* −, without NE challenge; +, with NE challenge

^f^sIgA = secretory immunoglobulin A

^g^All values represent the means with subtracted background OD_490nm_. Sera were diluted at 1:50 for the *C.perfringens* antigen detection, respectively, using ELISAs. Each sample was assayed in duplicate

^h^IAP = intestinal alkline phosphatase, μmol/mg protein

^i^SEM, standard error of the mean

^j^*P*-values represent the main effect of the diet, the main effect of NE challenge, and the interaction between the dietary treatments and NE challenge

### Anti-*C. perfringens* specific IgA and IgG concentration

No significant cooperative effect was observed between *B. coagulans* supplementation and NE infection in terms of serum specific IgG and jejunal specific secretary IgA of broilers (Table 7**)**. Compared to the uninfected birds, NE infection significantly (*P* < 0.05) resulted in an increase in the concentration of jejunal anti-*C. perfringens* specific IgA during the whole infection period, and sera anti-*C. perfringens* specific IgG at the early stage of infection (at 7 DPI, *P* = 0.038). In addition, *B. coagulans* inclusion resulted in higher (*P* = 0.008) anti-*C. perfringens* specific IgA concentrations in the jejunum at the early stage of infection, whereas significantly reduced serum specific IgG concentrations against *C. perfringens* at the later stage of infection (at 21 DPI, *P* = 0.031) relative to that in non-supplemented birds.

### *Mucin-2*, *LYZ*, *fowlicidin-2*, *TLRs,* and cytokine gene expression in the jejunum of broiler chickens

Changes in *TLR* mRNA expression in the jejunum are shown in Table 8. No significant interaction effect on *TLR2*, *TLR4*, cytokines, *mucin-2*, and *fowlicidin-2* mRNA expression was observed between NE infection and *B. coagulans* addition. Compared to uninfected birds, NE infection significantly (*P* < 0.05) decreased *mucin-2* (at 14 DPI), *TLR2* (at 7 and 14 DPI), *TLR4* (at 7 and 14 DPI), *TNFSF15* (at 7 and 14 DPI), *LYZ* (at 14 DPI), and *fowlicidin-2* (at 7 and 14 DPI) gene mRNA levels, whereas a significant (*P* = 0.001) increase in *IFN-γ* mRNA levels (at 7 DPI) was observed. The addition of *B. coagulans* significantly upregulated the expression of *LYZ* mRNA at 7 DPI (*P* = 0.011), whereas downregulated *IFN-γ* mRNA levels (at 14 DPI, *P* = 0.089) in contrast to that in the no *B. coagulans* group. On the other hand, no significant effects on *mucin-2, TLR2, TLR4*, and *TNFSF15* mRNA levels in the intestine of broilers during the whole experimental period were observed. NE-infected birds fed diets supplemented with *B. coagulans* showed an increase in *fowlicidin-2* mRNA levels (at 7 DPI, *P* = 0.057) compared to that in the NE-infected birds that did not receive any probiotics.

**Table 8:** Effect of *Bacillus coagulans* on immune-related gene expressions in the jejunum of broilers challenged with NE (n = 6)

Items	NE^c^	*MUC2*		*TLR2*		*TLR4*		*IFN-γ*		*TNFSF15*		*LYZ*		*Fowlicidin-2*	
		7 DPI^d^	14 DPI	7 DPI	14 DPI	7 DPI	14 DPI	7 DPI	14 DPI	7 DPI	14 DPI.	7 DPI	14 DPI	7 DPI	14 DPI
*Bacillus coagulans,* mg/kg															
0	–	1.17	0.79	1.01	1.07	1.04	1.19	1.06	1.01	1.01	1.18	1.16	1.10	1.07	1.53
0	+	1.02	0.36	0.48	0.30	0.62	0.43	2.36	1.22	0.58	0.25	0.76	0.25	0.85	0.54
400	–	1.47	0.94	0.90	1.05	0.99	1.12	0.95	0.86	1.09	1.35	1.65	0.84	1.70	1.79
400	+	1.09	0.49	0.50	0.46	0.39	0.62	2.58	0.74	0.79	0.39	1.71	0.46	0.64	1.13
SEM^e^		0.101	0.077	0.058	0.054	0.053	0.120	0.206	0.089	0.074	0.070	0.133	0.072	0.107	0.188
Main effect															
400		1.28	0.71	0.70	0.76	0.69	0.87	1.77	0.80	0.94	0.87	1.68^a^	0.65	1.17	1.46
0		1.09	0.57	0.74	0.69	0.83	0.81	1.71	1.12	0.79	0.72	0.96^b^	0.67	0.96	1.04
Challenged		1.05	0.42^b^	0.49^b^	0.38^b^	0.50^b^	0.52^b^	2.47^a^	0.98	0.68^b^	0.32^b^	1.23	0.36^b^	0.74^b^	0.83^b^
Non-challenged		1.32	0.86^a^	0.95^a^	1.06^a^	1.01^a^	1.15^a^	1.01^b^	0.94	1.05^a^	1.27^a^	1.40	0.97^a^	1.39^a^	1.66^a^
*P-*values^f^															
*Bacillus coagulans*		0.370	0.367	0.714	0.531	0.191	0.804	0.892	0.089	0.335	0.289	0.011	0.870	0.335	0.268
Challenged		0.204	0.008	<0.001	<0.001	<0.001	0.014	0.001	0.804	0.020	<0.001	0.527	<0.001	0.006	0.036
Challenged × Bacillus coagulans		0.562	0.935	0.594	0.397	0.393	0.603	0.679	0.364	0.670	0.924	0.393	0.114	0.057	0.671

^a,b^Means within the same column with different superscripts differ significantly (*P* < 0.05)

^c^NE = Co-challenged with *Eimeria* spp. and *Clostridium perfringens;* −, without NE challenge; +, with NE challenge

^d^DPI = days post infection

^e^SEM, standard error of the mean

^f^*P*-values represent the main effect of the diet, the main effect of NE challenge, and the interaction between the dietary treatments and NE challenge

## Discussion

BWG and lesion scores are commonly used as clinical measurements for evaluating the severity of NE. In the present study, reduced BWG and AFI and increased intestinal lesion scores were observed in NE-challenged birds. These indicated that NE infections led to inflammatory responses in the gut and negatively influenced growth performance. However, for the NE-infected birds, BWG and lesion scores significantly improved with *B. coagulans* supplementation compared with that in the control diet group. In addition, dietary *B. coagulans* supplementation remarkably increased BWG and improved FCR compared with those in the non-supplemented group. These results are in line with findings of most other studies that show that the use of the probiotic *B. coagulans* positively influences the growth performance of broilers [18–21]. The observed improvements in BWG and FCR may be attributable to the increase in feed consumption and improved nutrient digestibility due to enhanced endogenous enzyme secretion [20]. Furthermore, the improvement in growth performance after *B. coagulans* supplementation may also be due to the ability to produce exoenzymes such as amylase, protease, and lipase, vitamins, amino acids, and some unknown growth-promoting factors by directly fermenting food in the gut [19–21], which in turn stimulates small intestine peristalsis, boosts feed digestibility and availability, and promotes gut health [33, 47].

Intestinal morphology, including villus height and crypt depth, as well as the villus height to crypt depth ratio, intestinal microbiota balance, and intestinal bacterial translocation, are important indexes of intestinal health, recovery, and function. In the present study, birds infected with NE showed shorter villi height and decreased villus height to crypt depth (V/C) ratio in the jejunum, severe gut lesion scores in the small intestine, notable bacterial dysbiosis in the cecal contents, and higher level of *C. perfringens* invasion in the liver compared with that in uninfected birds, thereby suggesting that NE infection disrupts gut barrier integrity and intestinal microbial balance. However, the addition of *B. coagulans* in the broilers’ diet resulted in a relatively normal morphological structure in the jejunum, regardless of NE challenge. In addition, supplementation of *B. coagulans* to the NE-infected birds also resulted in a significant reduction of gut lesion scores in the small intestine, better balance of intestinal microflora, and lower *C. perfringens* load in the cecal contents and liver compared with those in the challenged birds that did not receive any probiotics. In line with our results, some studies have reported that *B. coagulans* improved the ecosystem of the intestinal tract in chickens [21], pigs [17], and humans [25]. Furthermore, previous studies have also suggested that *B. coagulans* improves gut health and reduces gut inflammation [30, 32]. Therefore, the results obtained herein imply that *B. coagulans* has the ability to maintain a mature and functionally active epithelium regardless of the NE challenge. These results also indicate that *B. coagulans* supplementation is effective in mitigating performance decline and gut lesions associated with NE, possibly by improving gut integrity and intestinal morphology as well as intestinal microflora balance in broiler chickens. The observed maintenance of gut barrier integrity due to *B. coagulans* supplementation is mainly attributable to the restoration of the balance of gut microbiota as well as to a reduction in pathogen toxin production by generating the antibacterial bacteriocin, proteases, and short-chain fatty acids [34, 38, 39, 41]. Another mechanism underlying the reduction in intestinal lesion scores due to *B. coagulans* supplementation may be the inhibition of intestinal proinflammatory cytokine overproduction or an increase in the production of anti-inflammatory cytokines [25, 26, 30, 32].

The avian innate immune system comprises a wide spectrum of defense mechanisms against invading pathogens and consists of a variety of immune effector cells, enzymes, proteins, and peptides.

Mucin-2 is mainly secreted by gut goblet cells, which are involved in the formation of an intestinal protective barrier that prevents intestinal bacterial translocation and maintains gut homeostasis [48]. In the present study, NE-infected birds fed diets with *B. coagulans* displayed a higher number of goblet cells in the jejunum, whereas no significant effect on *mucin-2* mRNA expression was observed compared with that in NE-infected but un-supplemented birds. Previous studies have shown that probiotics positively modify mucin dynamics in chicken intestines [49]. This change in *mucin-2* gene expression could be associated with a balanced intestinal microflora. Although no significant change in *mucin-2* mRNA levels was observed in the present study, a significant change in the number of goblet cells also indicates that dietary inclusion of *B. coagulans* could protect intestinal epithelial barrier integrity from enteric pathogens’ adhesion and invasion and improve the gut health of broiler chickens challenged with NE.

IAP is an endogenous protein expressed by the intestinal epithelium that is also believed to play a vital role in maintaining gut homeostasis and health through interactions with resident microbiota, diet, and the gut [50, 51]. Loss of IAP expression or function is associated with increased intestinal inflammation, dysbiosis, injured intestinal barrier structure, bacterial translocation, and subsequent systemic inflammation [52]. Therefore, stimulation of IAP activity by dietary intervention is a goal for preserving gut homeostasis and health by minimizing low-grade inflammation. In the present study, a decrease in IAP activity in the jejunum was observed after NE infection relative to that in the control, which indicated that there was an impaired intestinal defensive response in the NE-infected birds. In contrast, enhanced IAP activity in the jejunum of NE-infected birds fed with *B. coagulans* was observed, implying that *B. coagulans* had the ability to ameliorate NE-caused intestinal inflammation as well as maintain gut health by stimulating intestinal IAP secretion [53].

LYZ is a 1,4-β-N-acetylmuramidase that enzymatically cleaves a glycosidic linkage in the peptidoglycan component of bacterial cell walls, which results in the loss of cellular membrane integrity and cell death [54]. *Fowlicidin-2*, a member of the cathelicidin family, is an important antimicrobial peptide that has been identified in chicken and has been shown to exert highly potent antibacterial and lipopolysaccharide (LPS)-neutralizing activities [55]. In the current study, NE infection significantly downregulated *LYZ* and *fowlicidin-2* gene expression, implying that birds were more prone to *C. perfringens* infection, as evidenced by the higher level of *C. perfringens* that was detected in the gut and liver of NE-infected birds. However, a remarkable upregulation in *LYZ* gene expression at the early stage of NE challenge and an increasing trend in intestinal *Fowlicidin-2* gene mRNA levels were observed in NE-infected birds fed with *B. coagulans* compared with un-supplemented birds and infected controls. *Bacillus*-based DFM have been reported to enhance innate immune function by promoting the synthesis of endogenous antimicrobial peptides in the gut [56, 57]. In addition, lower levels of cecal *C. perfringens* growth and liver invasion were also observed in NE-infected birds that were fed *B. coagulans*, suggesting that *B. coagulans* supplementation might be capable of enhancing innate immunity against NE in broiler chickens. Similar results were also reported by Lee et al. [58] in that *B. subtilis* could augment macrophage function in chickens by promoting serum NO production.

TLRs are a group of evolutionarily conserved membrane receptors broadly expressed on various innate immunity cells and non-immune cells, where they function as the primary sensors to initiate innate immune responses by responding to pathogen-associated molecular patterns (PAMP) from bacteria, viruses, fungi, or parasites. Furthermore, these play a crucial role in defense against pathogens by activating the transcription factor NF-κB signaling pathway and induction of inflammatory cytokines, such as tumor necrosis factor alpha (TNF-α), interleukins (IL-6, IL-1β, IL-8, and IL-12), interferon (IFN), and co-stimulatory molecules [59]. In the present study, NE infection significantly increased the proinflammatory cytokine *IFN-γ* mRNA levels compared with those in the unchallenged control, indicating that NE challenge leads to intestinal inflammatory responses. Interestingly, decreased *TLR2*, *TLR4*, and *TNFSF15* gene expression at the middle and later phases of NE infection and downregulated gene mRNA levels of *mucin-2*, *LYZ*, and *fowlicidin-2* at the later phase of NE infection were observed in the jejunum of NE-infected birds. This finding indicates that birds have higher susceptibilities to *C. perfringens* infection. However, some previous studies have shown that NE infection could induce the expression of *TLR2*, *TLR4,* and some inflammatory cytokines (*TNF-α, IFN-γ*) by activating the *TLR*-mediated signal pathway [60, 61]. The observed decrease in the effect of NE infection on the expression of TLRs and some inflammatory cytokines may be attributable to different sampling time points and decreased pathogen load in the gut. Unknown factors that may be relevant here include the relative kinetics of the expression of individual cytokines, their sites of production within the intestinal tract, and the relative activities of these immune mediators during avian NE disease.

However, no significant effects on *TLR2*, *TLR4* and *TNFSF15* mRNA levels in the intestines of *B. coagulans*-fed birds were observed during the whole experimental period compared with those in the controls. This result showed that *B. coagulans* has an anti-inflammatory capacity and does not regulate *TLR*-mediated signal pathway responses in the intestine or its ability to inhibit the adhesion and liver invasion of other gut microflora, particularly some pathogenic bacteria that compete for available nutrients without decreasing the growth performance of chickens. The downregulated expression of the proinflammatory cytokine IFN-γ in the intestines of *B. coagulans*-fed chickens following NE infection indicates that *B. coagulans* decreases NE-induced intestinal inflammation. Similarly, several previous investigations have shown that *B. coagulans* reduces intestinal inflammation in mice by modulating intestinal cytokines’ profiles [28, 29, 32, 62]. Thus, the effect of *B. coagulans* supplementation on decreasing *IFN-γ* mRNA following NE infection could either reflect the alleviation of NE-induced damage or increase the ability of the host in clearing the pathogen *C. perfringens*. In addition, it is also possible that the reduced intestinal inflammatory response caused by *B. coagulans* could lead to an increase in the intestinal health of broiler chickens, thereby contributing to an improvement in growth performance.

Serum circulating IgG and intestinal secretory IgA levels against α-toxin and NetB toxin protect the host against *C. perfringens* infection [63]. Previous studies have indicated that Bacillus spp. could modulate chicken systemic or intestinal mucosal immunity [64–68]. In the current study, NE-infected birds fed *B. coagulans*-supplemented diets showed higher anti-*C. perfringens* secretory IgA antibody titers in the jejunal mucosa during the early phase of infection compared with those in NE-infected but non-supplemented birds. This increased expression in sIgA in the intestinal tract was accompanied by a decrease in intestinal *C. perfringens* colonization and reduced liver bacterial invasion. Secretory IgA is a major component of the intestinal mucosal barrier and plays an integral role in intestinal protection [69]. Therefore, our results show that the addition of *B. coagulans* protects intestinal mucosal surfaces from colonization and invasion of pathogenic microorganisms by stimulating intestinal mucosal B lymphocytes to switch into plasma cells for IgA synthesis at the early infection stage. However, at the later infection period, reduced serum anti-*C. perfringens* IgG antibody concentrations and no significant effect on intestinal sIgA levels in *B. coagulans*-supplemented birds were observed compared with those in the non-treated control. These findings may be due to a reduction in the number of *C. perfringens* in the liver of NE-infected chickens fed *B. coagulans* at the later infection stage, which was insufficient to stimulate the immune system to produce more specific IgG. Lee et al. [58] reported that *B. subtilis* had no significant effect on serum antibody levels against *C. perfringens*, implying that the continuous addition of dietary *B. coagulans* decreased the host immune inflammatory responses induced by *C. perfringens*, as a consequence of the exclusion of potentially harmful microbiota by *B. coagulans* during the later infection period.

## Conclusions

The present study demonstrated that *B. coagulans* supplementation is apparently effective in preventing NE occurrence and reduces the severity of *C. perfringens*-induced NE in broiler chickens. The protective effects of dietary *B. coagulans* supplementation on NE challenge was evidenced by an enhancement in innate and acquired immunity, suppression of pathogen colonization and invasion, and improvement in intestinal barrier function. Further studies are warranted to ascertain the cellular and molecular mechanisms used by *B. coagulans* in enhancing the innate immunity of chickens against NE infection.

## Abbreviations

ADFI: Average daily feed intake
ADG: Average daily gain
AMP: Antimicrobial peptides
BWG: Body weight gain
CD: Crypt depth
CFU: Colony-forming unit
ELISA: Enzyme-linked immunosorbent assay
FCR: Feed conversion ratio
GAPDH: Glyceraldehyde-3-phosphate dehydrogenase
IAP: Intestinal alkaline phosphatase
IFN-γ: Interferon-γ
IgG: Immunoglobulin G
LYZ: Lysozyme
NE: Necrotic enteritis
NRC: National Research Council
sIgA: Secretory immunoglobulin A
TLR: Toll-like receptor
TNFSF15: Tumor necrosis factor superfamily 15
V:C ratio: The ratio of villus height to crypt depth
VH: Villous height
